# Understanding profound autism: implications for stigma and supports

**DOI:** 10.3389/fpsyt.2024.1287096

**Published:** 2024-01-22

**Authors:** Elaine B. Clarke, James B. McCauley, Amy Lutz, Marina Gotelli, Stephen J. Sheinkopf, Catherine Lord

**Affiliations:** ^1^Department of Psychiatry and Biobehavioral Sciences, University of California, Los Angeles, Los Angeles, CA, United States; ^2^Department of Psychology, St. Mary’s College of California, Moraga, CA, United States; ^3^Department of History and Sociology of Science, University of Pennsylvania, Philadelphia, PA, United States; ^4^Fundación Brincar por un Autismo Feliz, Buenos Aires, Argentina; ^5^Consejo Nacional de Investigaciones Científicas Y Técnicas, Buenos Aires, Argentina; ^6^Thompson Center for Autism and Neurodevelopment, University of Missouri, Columbia, MO, United States

**Keywords:** autism spectrum disorder, profound autism, stigma and awareness, prevalence, mixed method, autistic adults, qualitative interview analysis

## Abstract

**Introduction:**

Societal perceptions and lack of understanding of autism spectrum disorder can be stigmatizing for autistic individuals and their families. This may be particularly the case for individuals who meet criteria for profound autism. Despite the considerable service needs of this marginalized group, there is little data on the prevalence of profound autism, nor on the experiences of those with profound autism and their families.

**Methods:**

The current study leveraged a mixed-methods approach to address these gaps. First, the prevalence of profound autism was examined in six samples—three from the United States and three from Western Europe. Second, inductive thematic analysis was used to code interviews from 20 caregivers of profoundly autistic adults.

**Results:**

The prevalence of profound autism varied widely across the six samples—from 11% to 48%. There were also notable differences between samples in prevalence by gender, race, and ethnicity. Two overarching themes were identified via inductive thematic analysis: *Community Perceptions of Autism and Family Support Needs and Advocacy Challenges*. Though caregivers were not directly asked about stigmatization during interviews, 85% of caregivers reported at least one instance of perceived stigma.

**Discussion:**

Future research should continue to examine the unique needs and stigmatization experiences of profoundly autistic individuals and their families across the life course.

## Introduction

Autism spectrum disorder (ASD), is a neurodevelopmental condition characterized by symptoms in two core domains: social communication difficulties (e.g., deficits in social–emotional reciprocity, limited use of eye contact, facial expressions and gestures, and difficulty maintaining relationships) and restricted, repetitive interests and behaviors (e.g., stereotyped motor movements, insistence on sameness, unusual and/or circumscribed interests, and unusual sensory interests) (1). Some individuals with ASD have average or above average IQ scores and strong language abilities. In contrast, others with autism have co-occurring Intellectual Disability (ID), limited or no use of spoken language, and require 24-h support. Importantly, the heterogeneity of autism is associated with differing needs, challenges, and strengths for individuals with autism and their families, including experiences of stigmatization.

The general public’s understanding—or lack thereof—of the social communication, behavioral, and other differences associated with autism can be stigmatizing both for individuals with autism and for their families. Erving Goffman famously defined stigma as “an attribute that is deeply discrediting” (2). In other words, stigma encompasses disapproval of and discrimination against individuals who are perceived to meaningfully differ from societal norms. Goffman’s argument that stigma could extend from those with “spoiled identities” to their families through what he called “courtesy stigma” has been used by many researchers as a springboard to the analysis of stigma experienced by parents of autistic children (3–6). One extreme example of autism courtesy stigma is the infamous “refrigerator mother” theory, in which parents of autistic individuals were thought to cause symptoms of autism through cold and insufficient caregiving (7). During the 1960s and 1970s—a period when there was virtually no empirically-based understanding of autism etiology—the refrigerator mother theory became widely accepted within the medical establishment, effectively labeling parents as the ultimate “scapegoats” for their children’s challenges (8).

Experiences of stigmatization may be especially pronounced amongst individuals who meet categorization criteria for profound autism (i.e., having either substantial intellectual disability, no or very limited language, or both, after age eight; see *A Note on Terminology*, below) and their caregivers, given the nature and severity of impairment inherently associated with these criteria. On a basic level, we need to know what proportion of individuals with autism fit these criteria to understand how many individuals with autism and their families may be at risk for experiencing stigmatization due to symptoms of profound autism. Because many profoundly autistic people cannot easily advocate for themselves, we also need to engage directly with caregivers to understand the stigmatization experiences of these individuals and their families, and to establish research priorities for this vulnerable group.

### A note on stigma

Scambler and Hopkins (9) clarified the difference between “felt” and “enacted” stigma: felt stigma describes the internalized negative feelings of the stigmatized, whereas enacted stigma refers to discrimination experienced by the stigmatized. From qualitative and quantitative studies, autistic individuals who are capable of advocating for themselves and their families have frequently expressed perceptions of both felt and enacted stigma based on their diagnosis, behaviors, language, or cognitive ability [for a review, see (10)]. These experiences can have notable impacts on mental health. Whereas reports of felt stigma—such as the embarrassment parents may conceivably feel when their autistic children have meltdowns—are rare, reports of enacted stigma are ubiquitous among caregivers of individuals with autism (3). Autistic children, particularly those with challenging behaviors, are implicitly and explicitly excluded both from private spaces, like family celebrations, and more public settings, such as restaurants, classrooms, and extracurricular activities (11–13). Further, caregivers of profoundly autistic adults—after decades of stigmatization and other negative experiences, and often in the face of limited adult services—may reduce efforts to find or create spaces in which their profoundly autistic loved one will be included (6). In short, enacted stigma is a considerable and ongoing challenge for profoundly autistic individuals and their families.

The lack of consideration of what constitutes appropriate supports to meet the needs and preferences of profoundly autistic individuals is another common form of stigmatization. The deinstitutionalization movement that began more than 50 years ago has had a tremendous effect on the types of services available to both autistic children and adults. A pervasive belief is that inclusive settings are always best for all disabled people, though empirical evidence supporting this view has focused on verbal autistic individuals with average or better cognitive abilities (14, 15). This bias has resulted in the shuttering of disability specific programs more broadly, from educational, to vocational, to residential settings (16). Inclusive options are appropriate for many autistic children and adults, who should, without question, be provided with whatever supports they need to thrive in the community. However, profoundly autistic individuals with severe cognitive impairments and sometimes dangerous behaviors–including aggression, self-injury, property destruction, pica, and elopement–may require structure, targeted instruction and behavioral support that can best be provided in intensive, disability-specific settings (17–19). The closure of many such programs has left families in crisis: parents repeatedly called to pick up their children at schools that cannot safely manage their behaviors; adults unable to obtain residential placement outside their parents’ home because agencies are financially incentivized to choose the easiest clients; overburdened and unequipped emergency rooms struggling to manage the growing influx of profoundly autistic individuals with nowhere else to go (20, 21). Families can feel that their options for support are limited and may feel obligated to continue as 24/7 primary caretakers. While there are reported emotional and relational benefits to arrangements of extended care in the family home for autistic adults (18), some families report exhaustion, stress, helplessness, and social exclusion arising from the challenges of caregiving (22). In short, challenges associated with the stigmatization experiences of families caring for individuals with profound autism warrant further study.

### A note on terminology

Diagnostic criteria for autism, particularly for subgroups or levels within autism, have changed several times in the history of this condition, with controversy for every attempt to subclassify what we now call ASD. These classification challenges and pursuant controversies are in part due to the heterogeneity seen in the presentation of autism. In efforts to reflect this, the 5th edition of the Diagnostic and Statistical Manual of Mental Disorders (DSM-5) and the 11th revision of the International Classification of Diseases (ICD-11), include specifiers and subcategories of autism. However, these classifications are rarely used in practice or in research (23). For example, DSM-5 introduced severity levels intended to indicate the degree of support required for individuals diagnosed with autism (1). These severity levels were not empirically validated, and in the years since their introduction, have not been consistently applied, though some school systems and insurance companies require their application (24, 25). Thus, challenges remain in parsing the autism spectrum to effectively indicate the capabilities and support needs of autistic individuals.

One recent effort to parse the differing needs, challenges, and strengths seen across the autism spectrum came from a commission from *The Lancet* on clinical research and care for ASD (23). Three authors of the current study (JM, MG, and CL) were also authors on *The Lancet* commission. *The Lancet* commission proposed a new term, profound autism, for individuals having either substantial intellectual disability (e.g., an intelligence quotient below 50), no or very limited language (e.g., limited ability to communicate to a stranger using comprehensible sentences), or both, after age eight, in addition to meeting criteria for autism. Given the wide range of needs of autistic people, the intent behind this term was to provide a clinically relevant way to identify autistic individuals who fit this profile to ensure their needs are not forgotten. Inherently, the nature and severity of impairment associated with profound autism criteria may place these individuals and their families at greater risk of experiencing stigma and marginalization.

New prevalence estimates from the Centers for Disease Control (CDC) found that over a quarter, 26.7%, of children with autism in the United States meet criteria for profound autism (26), suggesting that in the United States, a substantial minority of children with autism may be at increased risk of experiencing stigma due to the nature and intensity of their autism symptoms. Additional work is needed to further understand the prevalence of profound autism, particularly in clinical and community-based samples, so we can better understand what proportion of autistic individuals and their families may experience stigma related to profound autism. Such information is also critical to improving service planning for this population, as most profoundly autistic individuals cannot speak for themselves and are likely to need intensive support services for much or all their lives.

Prior to *The Lancet* commission, the term “profound autism” had been used by stakeholders, therapists, and researchers to broadly describe autistic individuals whose ability to live independently was significantly compromised [see (27)]. The term “severe autism” has also been used to similar effect (28). As part of *The Lancet* commission, a consensus of researchers and stakeholders—including autistic self-advocates and parents—proposed profound autism as an administrative term to clearly and efficiently indicate individuals with autism who need extensive functional assistance, specifically, “requiring 24 h access to an adult who can care for them if concerns arise, being unable to be left completely alone in a residence, and not being able to take care of basic daily adaptive needs.” [(23), p. 278].

There is disagreement in scientific and advocacy communities about the choice of the term profound autism, with some arguing it is “misleading and counterproductive” [(29), p. 94] to efforts of the neurodiversity movement to conceptualize and advocate for autism as an identity, not a disabling medical condition [see also (30)]. However, clear terms are needed to describe the extensive clinical needs of profoundly autistic individuals and their families. Reliable terminology is also necessary to support high-quality empirical investigations necessary to better understanding and supporting profoundly autistic individuals. With precise terminology, we can also start to develop and apply standard methodology for classification. This is an ongoing effort, particularly when classifying adults, because available measures for IQ and autism classification are typically adapted from assessments for younger children (31). Despite the controversy, the term profound autism has already been applied in a number of research studies [see (26, 32–35)].

The proposal of the term profound autism and subsequent debate has highlighted the current cultural politics of autism. The rise of the neurodiversity movement has changed how autism is discussed and conceptualized, both in public and academic circles, to emphasize the experiences and opinions of autistic individuals who speak for themselves. But the priorities, service needs, and life experiences, including experiences of stigmatization, of autistic self-advocates are frequently quite different from those of profoundly autistic individuals and their families. It is essential that elevating the perspectives of autistic self-advocates does not come at the cost of amplifying the stigmatization experienced by profoundly autistic people, who are often not capable of self-advocacy, as well as their families. Ultimately, the agreed-upon term to describe this subgroup of individuals with ASD is far less important than acknowledgement that this group has extensive service and daily life support needs—needs which are often not adequately met by existing services—and that the nature of profound autism puts these individuals and their families at increased risk of experiencing marginalization and stigma.

### The current study

The current project consisted of two distinct but related goals. First, to better understand what proportion of individuals with autism may be at risk of experiencing stigma related to meeting profound autism criteria, we wanted to establish the prevalence of profound autism in a range of samples from the United States and Western Europe. Second, to better understand the stigmatization experiences of individuals with profound autism and their families, we wanted to directly ask caregivers of individuals with profound autism about their life and stigmatization experiences. Thus, the aims of this study were as follows:

1. To establish the prevalence of profound autism in six autism cohorts, three from the United States and three from Western Europe, and to examine variation in prevalence estimates by gender, race and ethnicity.
2. To qualitatively examine experiences of stigmatization, challenges, and service needs reported by caregivers of adults with profound autism in varying regions of the United States.
  Given the disparate approaches required to accomplish each of these two aims, the method and results for Aims 1 and 2 are reported separately.

## Aim 1: profound autism prevalence estimates

### Method

#### Autism cohorts

Prevalence estimates of profound autism were calculated by gender, race, and ethnicity in six samples, three from the United States and three from Western Europe. All data included in the current project was de-identified, and all six studies were approved by their respective institutional review boards (IRBs). The U.S. samples included the Early Diagnosis (EDX) cohort, the Adolescents and Adults with Autism (AAA) sample, and the Rhode Island Consortium for Autism Research and Treatment (RI-CART) sample. The EDX cohort was initially recruited in the early 1990s and consists of 192 consecutive referrals to community-based clinics in North Carolina (58%) and the greater Chicago area (42%). Participants were initially seen between the ages of 2 and 3—data for the current analyses was collected when participants were approximately age 9. The AAA sample, initially recruited in the late 1990s, consists of 406 individuals with a preexisting diagnosis of ASD or a related condition (i.e., Asperger’s Syndrome, Pervasive Developmental Disorder, Not Otherwise Specified [PDD-NOS]) aged 10 or older at the time of recruitment and assessment. Half the sample (49.6%) was recruited from Wisconsin, and the remaining half (50.4%) was recruited from Massachusetts. RI-CART is statewide community-based sample of individuals with autism living in Rhode Island and surrounding geographic regions [i.e., Southeastern Massachusetts, Northern Connecticut; (36)] initially recruited in the 2010s. Individuals of all ages who had a preexisting autism diagnosis or who were suspected of meeting criteria for an autism diagnosis by a community provider or family member were eligible to participate. 1,016 individuals who participated in RI-CART between the ages of 8 and 25 are included in the present study.

The Western European samples included the UK-based QUEST sample and the Special Needs and Autism Project (SNAP) cohort, and the Norwegian Mother, Father, and Child (MoBa) cohort. All three samples were initially recruited in the mid 2000’s. QUEST is a community-based sample of 277 children living in one of two districts in London, Bromley and Lewisham (37). Notably, girls with autism were over-sampled within QUEST to allow for more robust sex comparisons (38). QUEST data for the current analyses were collected when participants were approximately age 13 (38). SNAP is a population-based study drawn from an initial cohort of 56,946 children living in South Thames, United Kingdom. A weighted epidemiological design was used to target a subsample of children considered most at risk for autism [see (39)]. A stratified subsample of 255 children (223 males) completed comprehensive diagnostic, IQ, and language assessments at approximately age 12 (40). Led by the Norwegian Institute of Public Health, MoBa is a population-based pregnancy cohort of 114,000 children born between 1999 and 2009. MoBa data for the current analyses were collected when participants were approximately age eight (41). Preliminary prevalence data from three of these samples (EDX, MoBa, SNAP) was included in *The Lancet* commission (23). Summary information on all six samples is included in Table 1.

**Table 1:** Demographic characteristics of the six samples.

Sample	Geographic location	*n*	Sex		Race				Ethnicity		Caregiver native language^a^
			Male	Female					Non-Hispanic	Hispanic	
**Adolescents and Adults with Autism (AAA)**	Wisconsin, Massachusetts	406	297	109	**White**		**Black**	**Other**	398	8	–
					376		10	12			
**Early Diagnosis Cohort (EDX)**	North Carolina, Illinois, Michigan	192	158	34	**White**		**Black**	**Other**	188	2	–
					146		46	2			
**Rhode Island Consortium of Autism Research and Treatment (RI-CART)**	Rhode Island	1,016	795	221	**White**	**Black**	**Multi**	**Other**	740	131	856 English 46 Spanish 2 Portuguese 4 Other 106 Missing/not reported
					722	34	81	47	145 missing or not reported		
					132 missing or not reported						
**Norwegian Mother, Father, and Child Cohort (MoBa)**	Norway	188	146	42			–		–		Norwegian: 144
											Not Norwegian: 40 4 Missing/not reported
**Special Needs and Autism Project (SNAP)**	South Thames, United Kingdom	155	139	16	**White**		**POC**		–		–
					147		8				
**QUEST**	London, United Kingdom	80	46	34	**White**	**Black**	**Multi**	**Other**	–		–
					38	22	10	9			

a For the MoBA sample only, caregiver native language was used as a proxy for measuring racial and ethnic diversity.

#### Phenotypic characteristics

Individuals were considered as meeting criteria for profound autism if at age eight or older they had an IQ score of less than 50 and/or little to no spoken language. In the EDX, RI-CART, MoBa, QUEST, and SNAP samples, individuals were classified as minimally or nonverbal if they were administered an Autism Diagnostic Observation Scale [ADOS; (42)] Module 1 at or after age eight. In the AAA sample, individuals were classified as minimally or non-verbal based on scores from item 33 (Overall Level of Language) on the Autism Diagnostic Interview [ADI; (43)]. The EDX, MoBA, SNAP, and QUEST samples administered IQ assessments chosen from standardized hierarchies based on child age and ability at the time of assessment [see (40, 44, 45), and (46), respectively, for information on the specific IQ measures used in each sample]. In the RI-CART sample, IQ was determined via scores on the Kaufman Brief Intelligence Test, Second Edition [KBIT-2; (47)]. In the AAA sample, IQ was determined via scores on the Wide Range Intelligence Test [WRIT; (48)] and/or maternal report (49).

#### Demographic characteristics

Given the various geographic locations and time periods that participants were recruited, the proportion of participants from racially and ethnically diverse backgrounds, and the criteria used to classify participants as racially and/or ethnically diverse differs considerably across the six samples reported here. The proportions of male and female participants within each sample are also quite variable. In the United States, the EDX sample was 76% White, 23% Black, and 1% Other (1 Asian participant and 1 American Indian participant). Only 2% of EDX participants identified as Hispanic. Males comprised 82% of the EDX sample. The AAA sample was 94% White, 3% Black, and 3% Other (6 Asian participants, 2 American Indian, and 4 Other). Two percent of AAA participants identified as Hispanic. Males comprised 73% of the AAA sample. Finally, the RI-CART sample was 71% White, 3% Black, 8% Multiracial, and 5% Other, with 13% of the RI-CART sample not reporting their race or missing information on race. For ethnicity, 13% of the RI-CART sample identified as Hispanic, 73% identified as non-Hispanic, and 14% of the sample chose not to report their ethnicity or were missing information on ethnicity. Additionally, 84% of the RI-CART sample identified the native language of the primary caregiver as English, 5% Spanish, 0.6% Other, and 10% of the sample chose not to report caregiver native language or were missing that information. Males comprised 78% of the RI-CART sample (Table 1).

In Western Europe, the QUEST sample was 48% White, 28% Black African or Black Caribbean, 13% Multiracial, and 11% Other. QUEST did not collect information on participants’ ethnicity, nor did SNAP or MoBa. Males comprised 58% of the QUEST sample. The SNAP sample was 95% White and 5% people of color. Ninety percent of the SNAP sample was male. Finally, the MoBa sample did not collect information on participants’ race but did ask about the primary caregiver’s native language. To be able to participate in MoBA, primary caregivers had to be able to read in Norwegian (41). Seventy-seven percent of the MoBa sample identified Norwegian as the native language of the primary caregiver, 21% identified a language other than Norwegian as the native language of the primary caregiver, and 2% of the sample chose not to report or were missing information on the primary caregiver’s native language. Males comprised 78% of the MoBa sample (Table 1).

#### Analytic plan

The cohorts included in this project used different methods of sampling, recruitment, and behavioral assessment. Notably, only two of these samples, MoBa and SNAP, are population-based. Thus, prevalence estimates for each of the six samples were calculated and are reported separately. Prevalence estimates for profound autism and corresponding confidence intervals were calculated by sex and race/ethnicity. For samples that had limited numbers of racially and ethnically diverse participants (AAA, SNAP) or, the majority of racially and ethnically diverse participants fell into a single racial category (Black—EDX), prevalence estimates are reported for white participants and participants of color. Because MoBa did not collect data on participant race and ethnicity, prevalence estimates are reported based on the native language of the primary caregiver (Norwegian/not Norwegian) instead. Both QUEST and SNAP provided comprehensive autism diagnostic and cognitive assessments to stratified subsamples—given this, both weighted and unweighted prevalence estimates and confidence intervals are reported for QUEST and SNAP (Table 2). Data management and analysis were conducted using Stata 17 and R version 4.3.0 (50).

**Table 2:** Profound autism prevalence estimates by sample, gender, and race.

Sample		Profound autism prevalence						
		Overall	Gender		Race			
			Male	Female	White	People of color^a^		
**Adolescents and Adults with Autism (AAA)**		57% (49 – 64%)	54% (45 – 62%)	70% (51 – 84%)	52% (42 – 61%)	69% (55 – 81%)		
**Early Diagnosis Cohort (EDX)**		48% (37 – 58%)	4% (0 – 11%)	23% (10 – 36%)	34% (27 – 42%)	70% (55 – 81%)		
**Special Needs and Autism Project (SNAP)**	**Unweighted**	23% (16 – 30%)	22% (16 – 30%)	25% (7 – 52%)	22% (16 – 30%)	20% (10 – 37%)		
	**Weighted**	20% (10 – 36%)	21% (10 – 39%)	15% (3 – 50%)	25% (3 – 65%)	11% (1 – 55%)		
**QUEST**	**Unweighted**	31% (21 – 43%)	26% (14 – 41%)	14% (7 – 26%)	**White**	**Black**	**Multi**	**Other**
					29% (16 – 30%)	45% (24 – 68%)	10% (0 – 45%)	33% (7 – 70%)
	**Weighted**	18% (11 – 28%)	38% (22 – 56%)	38% (23 – 56%)	15% (7 – 29%)	30% (14 – 55%)	6% (0 – 44%)	23% (4 – 69%)
**Rhode Island Consortium of Autism Research and Treatment (RI-CART)**		11% (8 – 15%)	14% (10 – 19%)	9% (4 – 17%)	**White**	**People of color** ^**a**^		
					13% (9 – 18%)	16% (11 – 22%)		
**Norwegian Mother, Father, and Child Cohort (MoBa)**		18% (12 – 24%)	17% (12 – 24%)	45% (28 – 63%)	**Caregiver native language** ^**b**^			
					**Native Norwegian speaker**	**Non-native Norwegian speaker**		
					23% (17 – 30%)	22% (11 – 39%)		

a Due to limited numbers of racially and ethnically diverse participants (AAA, SNAP) or the majority of racially and ethnically diverse participants belonging to a single racial/ethnic group (Black, EDX), racial and ethnic prevalence estimates for these samples were collapsed into binary categories.

b For the MoBA sample only, caregiver native language was used as a proxy for measuring racial and ethnic diversity.

### Results

#### United States samples prevalence estimates

The proportion of individuals meeting one or both criteria for profound autism criteria was 57% (95% CI 49–64%) in the EDX sample. A higher proportion of females in EDX met profound autism criteria than males, although confidence ranges overlapped (70% vs. 54%, see Table 2 for confidence intervals). Moreover, a higher proportion of participants of color met criteria for profound autism in the EDX sample compared to white participants (69% vs. 52%). In the AAA sample, 35% (95% CI 29–42%) of participants met criteria for profound autism. The proportions of females and males who met profound criteria were quite similar, 37 and 35%, respectively. Whereas 20% of white participants in the AAA sample met profound autism criteria, only 10% of participants of color did, though confidence intervals overlapped (Table 2). Only 11% of the RI-CART sample met criteria for profound autism. A lower proportion of females met criteria than males, though again, confidence intervals overlapped (9% vs. 14%, Table 2). Thirteen percent of white participants in RI-CART met profound criteria, and 16% of participants of color met profound criteria—again, confidence intervals overlapped (Table 2).

#### Western Europe samples prevalence estimates

The weighted proportion of individuals meeting criteria for profound autism in the QUEST sample was 18%. Thirty-eight percent of both male and female participants in QUEST were classified as having profound autism. Higher proportions of Black African and Black Caribbean participants and participants who identified their race as Other (30% and 23%, respectively) met criteria for profound autism than white and multiracial participants (15 and 6%, respectively), though confidence intervals overlapped (Table 2). In the SNAP sample, the weighted proportion of individuals with profound autism was 20%. A lower proportion of females met criteria than males (15% vs. 21%), though confidence intervals overlapped (Table 2). A larger proportion of white participants in SNAP met profound autism criteria than participants of color, though again, confidence intervals overlapped (25% vs. 11%). Both weighted and unweighted prevalence estimates for the QUEST and SNAP samples are reported in Table 2. Finally, in MoBa, 23% of participants met one or both criteria for profound autism. A higher proportion of females met profound autism criteria than males, although confidence ranges overlapped (45% vs. 17%, Table 2). Similar proportions of MoBa participants whose primary caregiver was a native Norwegian speaker and participants whose primary caregiver was not a native Norwegian speaker met criteria for profound autism (23 and 22%, respectively).

## Aim 2: qualitative caregiver interviews

### Method

#### Participants

A total of 20 caregivers of autistic adults (average age of autistic adult = 24.6) agreed to be interviewed. The autistic adults were mostly male (*n* = 18) and most were white (*n* = 18). Participating families resided in a wide range of geographic regions in the US, including West (*n* = 6), Northeast (*n* = 5), South (*n* = 3) and Midwest (*n* = 5). Additionally, one participating family resided in Canada. The majority of autistic adults were living in the family home (*n* = 16), with the remainder living in residential care, group homes, or a combination of family home and residential care. Families were eligible to participate if they were parents or legal guardians of a child over 18 with a diagnosis of autism spectrum disorder, if the child either had a co-occurring diagnosis of intellectual disability, had minimal communication capabilities and/or required extensive daily assistance. Within recruitment materials, the phrase “Autistic Adults with High Support Needs” was used, and families within interviews mainly used the term autism, but a small number (*n* = 4) also used “profound autism” or “severe autism” to describe their adult children’s diagnosis and behavior.

#### Procedure

Caregivers of autistic adults were invited via social media to participate in interviews about their adult children’s needs for quality of life and their family’s needs and challenges related to caregiving an autistic adult. Purposive and snowball sampling techniques were used, specifically, posting flyers to community websites or groups specific to caregivers of autistic adults and by asking families to share the research flier with others. This study was approved by the Saint Mary’s College of California Institutional Review Board, and written consent was obtained from all caregivers. The interviews were all conducted remotely via Zoom and transcribed by research assistants. Interviews lasted approximately 55 min. Caregivers were asked about their needs, community perceptions, and their adult child’s needs in a semi-structured interview regarding quality of life. Some open-ended questions included, “How do people in your community view autism?,” “Do you easily find support in your community for disabilities or autism?,” and “Do you experience any negative reactions to autism in your community?.” For the current study, only themes regarding family needs and community perceptions from the interviews are reported to gain a nuanced account of perceived stigma and marginalization. These themes were largely reflected in participant responses to questions surrounding community experiences when their adult was present and current family support needs. The second author analyzed the data using inductive thematic analysis by applying codes to data, developing a codebook, and constructing themes and subthemes in an iterative process in collaboration with trained research assistants (51). After the development of themes, the dataset was analyzed again and subthemes were then refined, recategorized, and renamed.

#### Researcher positionality

It is important to acknowledge the positionality of the qualitative coders for this research, as such factors may influence the analysis and interpretation of qualitative data. Specifically, it is important to acknowledge how the perspectives of the researcher may differ from the participants and the reader, and that these might influence data collection or interpretation in subtle but meaningful ways [see (52)]. The initial motivation for this protocol was to understand the lived experiences of autistic adults and their families with a focus on understanding factors related to quality of life. Participants were informed of the second author’s experience with autism as a researcher before completing interviews. One research assistant involved in developing the qualitative portion of the current study identified as a sibling of individuals with profound autism.

### Results

A detailed description of the themes, subthemes, definitions, and examples is presented in Table 3. All excerpts presented have been anonymized.

**Table 3:** Caregiver perception and experience of stigma: themes, subthemes, definitions, and examples.

Themes	Subthemes	Definitions	Examples from transcripts
Community perceptions of autism	Perceived stigma	Caregiver reporting negative responses or interactions for community members witnessing behavioral problems in autistic adult at some point in development	“I’ve had people take video when he was having a meltdown in a public place. I’ve had people say that I wasn’t controlling him. I’ve had people say I wasn’t trying hard enough. I’ve had people saying I was trying too hard. It really runs the gamut.” “I’ve had a few people tell me there are institutions for people like her. You know, other people look at you like she’s some sort of alien. It’s when you find other parents who have children with special needs, that you get the nice smile than the Oh, do not worry about it. It’s okay. If she’s humming.” “I think we get more pity than, than, than people who are just unkind but there definitely are people you know, that stare and that, you know, aren’t curious. They’re just, they are muttering to themselves or whatever. They just want to, you know, if you are curious, fine, but just kind of, you know, the 20-year-old guy bouncing down the aisle in the supermarket wearing a Sesame Street shirt and knocking things over and bumping into people and stuff like that.”
	Incomplete knowledge of autism	Caregiver reporting perceptions of autism in the community that is not representative of individuals with lower cognitive abilities, high support needs, and/or with more behavioral concerns	“I think generally society has the wrong view of autism. They do not, they are not thinking about severe autism, like what [my son] has. They’re thinking about, you know, The Good Doctor. And that really leaves [my son] out of the conversation. And the politicians, they will not listen to us … They’ll only listen to the self-advocates; they will not listen to us. And, and that sort of like, that’s why I have to fight like hell for things that they should probably be giving me without a fight.” “I think a lot of people just have no idea what it is. Because, you know, we go for walks, and [my son] is very vocal, he jumps around, he makes fast movements. I cannot, I feel like I cannot really take him out in public. Like, because he just, he just freaks people out. He gets people in people’s face. And he’s really hard to control. And he’s bigger than I am. And he’s stronger than I am. And unless it’s an open space, it’s really hard to take him out much.” “People do not see behaviors as a part of autism. But unfortunately, it’s, it’s a very big part. It’s just the unspoken part, you know, you do not see, you know, the morning news shows really doing it, they do the segments of, you know, the kid with special needs, who was chosen homecoming king, or, you know, they do not do you know, the parents that get beaten up one minute, but then are like, loved and hugged on the next.”
	Attempts to build community	Caregivers describing attempts to establish community supports and routines for autistic adult	“I would say, we have tried to foster a community around him that’s really supportive. So, all of our neighbors know him. And everybody, you know, says hi to him, and everybody kind of looks out for him. And that, that goes into the general community, for instance, like when he where he shops, and where he recreates and things like that, everybody knows him. And so, they are all very supportive of him.” “I feel like it’s kind of, and we were lucky too that my, my daughter who has autism was fully included. And so, she kind of grew up in a neighborhood school with peers and her sister and you know, so I think people are a lot more accepting than maybe elsewhere. There’s definitely misconceptions about it. Definitely. When, when they were little, it was really hard to take them out in public, but we sort of have kind of worn people down, I think over like, 20 years, like, we do not get the same looks in church that we used to get, because we have been going for 20 years. So it’s kinda like, “oh, it’s them” you know, it’s just kind of that kind of thing.”
Family support needs and advocacy challenges	Limited support for housing and activities, and healthcare	Caregivers reporting difficulties establishing appropriate placements and activities	“And we have asked to have him placed in a group home, but he has not been placed yet. The stress for us, is very dependent on his behavior. But he is still, even when he’s having very good days, and he has many of those in a row, he’s still very limited on what he will let us do. I mean, he does not want to go out and shop or go to any activity that does not really involve food. So, his dad and I are stuck at home, he does not travel well, he does not fly well. He does not, you know, unless it’s Disneyland, he does not like to go anywhere.” “I mean, so it’s difficult, it really, we have had to sue and hire lawyers to get services and different places. You know, go to mediation, frequent meetings, that sort of thing. So, you really have to, we had to really fight for getting him services, and then we pay privately on our own when we did not feel like they were able to meet his needs.” “What I would say when I look at some of these kids who are on the severe end of the spectrum like my son that, you know, I think a lot of people underestimate our kids and what they are capable of, including families. And I think part of that is that the professionals sometimes have lower expectations, and so I think what’s really been important for us is to have these very high expectations and to always, you know, assume that [our son] can learn something, that he can do something.
	Frustration about health and healthcare experiences	Caregivers reporting difficulties finding competent healthcare services or challenging experiences with healthcare professionals	“It’s really hard dealing with healthcare professionals, and how little they know about this disability and, or the Americans with Disabilities Act.” “I think if I could do things differently over the years is that the medical professionals would do more tests instead of just saying, well, that’s cause she’s got autism, you know, and I mean, I think we put her through a lot of physical pain with, you know, the things that have been wrong with her physically, that she wasn’t able to tell us.”
	Inadequate services and staffing	Caregivers reporting understaffed placements, high turnover rates for staff, or difficulties finding adequate support personnel to accommodate needs	“Having to find a respite worker, there’s nobody, really. Nobody that can handle autism. And I would just not feel comfortable having a stranger come in … because he can get angry very fast, and if he gets angry, he will hurt you. And I feel like I cannot put somebody through that. I mean, I’d feel really bad about leaving it. And then if they did something accidentally, and that they did not even know when they made him mad accidentally, then you know, they are in danger.” “I’m just thinking lack of support as far as not being able to have enough staff to help us with him … having such a hard time finding staff since he’s graduated from high school.” “I could use another caregiver or two. But just have a hard time finding people, and then finding people that she would trust. She’s been through a lot. So she does not trust a lot of people.”

#### Community perceptions of autism

##### Perceived stigma

Caregivers reported a wide range of experiences in their communities and advocacy networks. Most notably, 85% of caregivers in the data reported some instance of perceived stigma, characterized by negative responses or interactions with community members. Of the individuals reporting stigma, many (*n* = 10/17) described a stigma event happening in childhood, with the remaining reporting instances of negative interactions in the community in the present day. Caregivers remarked about negative reactions from strangers within grocery stores, religious institutions, and other public locations. Other caregivers reported how others had negative reactions to the ways in which their children made noises, moved, or displayed aggressive behavior in public.

##### Incomplete knowledge of autism

Caregivers also reported hearing public conversations about autism that were not representative of individuals with lower cognitive abilities, limited communication, or behavioral concerns. Caregivers described frustration with social movements surrounding autism. For example, one mother of a son in his early twenties with profound autism stated:

I think generally society has the wrong view of autism. They don't, they're not thinking about severe autism, like what [my son] has. They're thinking about, you know, The Good Doctor. And that really leaves [my son] out of the conversation. And the politicians, they won't listen to us … They'll only listen to the self-advocates; they won't listen to us. And…that's why I have to fight like hell for things that they should probably be giving me without a fight.

Caregivers also said that public conceptions of autism did not consider behaviors such as intense vocalizations or aggression as being related to autism. Instead, caregivers reported members of the public were more familiar with higher cognitive abilities and/or extraordinary talents being associated with autism. For example, another mother of a son in his early twenties with profound autism explained that she had to correct people during community interactions: “They’ll often ask if he has some savant or particular talent.”

##### Attempts to build community

Caregivers talked about attempts to establish routines and trusted social networks for their children. Many caregivers reported that their children liked to be around peers with whom they were familiar and benefited from integration into community events. For example, one mother explained “I think over like, 20 years, like, we do not get the same looks in church that we used to get, because we have been going for 20 years. So it’s kinda [*sic*] like, ‘oh, it’s them.’”

#### Family support needs and advocacy challenges

##### Limited support for housing and activities

Caregivers described difficulties establishing appropriate activities or living situations for their adult children. Some families reported activities for their children had stopped because of the COVID-19 mitigation efforts, and that resumption of these activities was slow-going. One father explained that his son enjoyed walking around the community, but his residential program did not restart community outings for almost 2 years following COVID-19.

Many families also noted difficulties finding appropriate housing placements for their children or being on waitlists for residential care. One mother of a son in his early twenties with profound autism reported:

In order to even be able to apply in our state for supported living homes, we had to submit his application to group family homes and get denied. He received over a dozen denials in one week. And so as far as…the supported living homes, which are supposed to be for people like [our son], there’s so few of them, and so many people that want to get in, and the homes choose the people they take.

The need for better opportunities for both activities and being around peers was frequently reflected on by caregivers.

##### Frustration about health and healthcare experiences

Families also reflected on challenges working with physicians or other healthcare professionals. Some caregivers reported difficulties getting accommodations during medical care visits, such as the presence of support staff. Others reported encountering professionals with limited awareness of autism or associated behaviors. One mother expressed frustration trying to get medical attention for her daughter in her late thirties with profound autism due to her inability to communicate pain: “the medical profession does not look further than just saying, well, she has autism. So that’s why she has bad behavior.” Another mother of a son in his early thirties reported frustrating experiences, noting healthcare professionals frequently, “have had no training in autism, and they do not know how to accommodate [my son], or necessarily do they want to, or feel they need to.”

##### Inadequate services and staffing

Caregivers also described frustration finding appropriate staff for respite, in-home care, and/or high-turnover rates at day programs or residential programs. Some caregivers reported it was difficult to find staff that they and their adult children trusted. Others stated that they were worried what might happen when they were not present. For example, one mother of an adult in his early twenties noted both difficulties finding care staff and discomfort with leaving her son alone with unfamiliar staff:

“Having to find a respite worker, there's nobody, really. Nobody that can handle autism. And I would just not feel comfortable having a stranger come in … because [my son] can get angry very fast, and if he gets angry, he will hurt you. And I feel like I can't put somebody through that. I mean, I'd feel really bad about leaving it. And then if they did something accidentally, and that they didn't even know when they made him mad accidentally, then you know, they're in danger.”

Indeed, many families with an autistic adult living at home felt constrained in their ability to take breaks for themselves and to develop longer term plans for their children amid their own aging experiences without trusted support available.

## Discussion

Stigma impacts individuals across the autism spectrum. For those who meet criteria for profound autism, marginalization due to communication challenges and considerable daily care needs may lead to distinct stigmatization experiences. However, to date, there is a relative lack of research on this group of autistic individuals and their families; as discussed below, this is a particular issue within Low- and Middle-Income Countries (LMICs). To better understand profound autism, we first examined the prevalence of profound autism in six distinct samples, three from the United States and three from Western Europe. To examine how stigma impacts the daily life experiences of profoundly autistic individuals and their families, we then conducted qualitative analyses of interviews with caregivers of adults with profound autism.

Though prevalence estimates vary across the samples reported here, in all six cohorts, profound autism represents a sizable minority of autistic individuals. These samples were recruited at distinct points in time, ranging from the 1990’s (AAA, EDX), to the early 2000’s (SNAP, QUEST, MoBa) and the mid-to-late 2010’s (RI-CART). The samples initially identified three decades ago, in the 1990’s, had the highest prevalence of profound autism—48% in the EDX sample and 35% in the AAA sample, respectively. In contrast, in the Western European samples, which were initially identified in the mid 2000’s, the prevalence of profound autism hovered around 20% (QUEST, 18%; SNAP, 20%; MoBa, 18%). The most recently ascertained sample, RI-CART, had the lowest prevalence rate of profound autism—11%. Notably, RI-CART also differed from the other samples reported here in that it included individuals who received an autism diagnosis in adolescence or early adulthood as well as individuals diagnosed in childhood, as there was no age limit for joining the study (36). These results suggest that as the overall prevalence of autism spectrum disorder has increased, the relative proportion of autistic individuals meeting profound criteria has decreased (26, 53). In other words, individuals with fluent language and average or better cognitive abilities constitute an increasingly large portion of the autism population, at least in the United States and the United Kingdom. As access to assessment and treatment services and public awareness of autism has increased, identification of autistic individuals with relatively mild behavioral presentations has improved. This represents a substantial shift from the 1990’s and 1980’s, when it was widely accepted that at least half of people with autism spectrum disorder had a comorbid intellectual disability (40, 54).

A recent analysis from the CDC of population-based surveillance data collected between 2000 and 2016 found that approximately 27% of eight-year-olds with autism in the United States met criteria for profound autism (26). Notably, Hughes and colleagues found the prevalence of both autism spectrum disorder and profound autism increased from 2000 to 2016 (2023). However, the prevalence of autism spectrum disorder increased at a much faster rate—from 3.9 in 1000 children in 2000 to 14.3 in 1000 children in 2016—than the rate at which the prevalence of profound autism increased—from 2.7 in 1000 children in 2000 to 4.6 in 1000 children in 2016. These findings suggest that as clinical practice has evolved, the sensitivity for diagnosing individuals without significant cognitive or language delays has increased. The decrease in the relative proportion of autistic individuals meeting profound autism criteria may be an indirect result of this diagnostic shift.

In three of the six samples (AAA, EDX, MoBa), a higher proportion of females met criteria for profound autism than males, though confidence intervals overlapped. In two samples (RI-CART, SNAP) a higher proportion of males met criteria than females—though again, confidence intervals overlapped. Finally, in QUEST, an equal proportion of males and females met criteria for profound autism—importantly, females were over-sampled in the QUEST study (38). This contrasts with prior findings that females with autism are more likely to have comorbid intellectual disability and similar challenges than males (55–57) and differs from the recent CDC estimates, which found 31% of females met profound autism criteria, compared to 26% of males (26). Awareness of and diagnostic processes for autistic women and girls have changed markedly in recent years, with increasing numbers of females with average or better IQ and verbal abilities receiving ASD diagnoses, both in childhood and later in life (58–60). These shifts in the understanding of autistic women and girls may explain some of the differences seen across samples.

The prevalence rates by race and ethnicity also differed considerably across samples. In three of the six samples, a higher proportion of racial and ethnic minority groups (EDX, QUEST, RI-CART) met criteria for profound autism than white individuals, though confidence intervals for QUEST and RI-CART overlapped. In MoBa, the prevalence of profound autism between individuals whose primary caregiver was a native Norwegian speaker and individuals whose primary caregiver was not a native Norwegian speaker were almost the same, 23% for the former, 22%, suggesting that caregiver native language did not contribute to the likelihood of meeting profound autism criteria. A higher proportion of white participants met profound autism criteria in AAA and SNAP, though again, confidence intervals overlapped. Of the samples reported, AAA and SNAP had the fewest racially and ethnically diverse individuals (comprising 7 and 5% of all participants, respectively)—this may have contributed to the lower prevalence rates seen here. AAA was a convenience and volunteer sample, which may have contributed to the limited representation of minority families.

These results do not provide conclusive evidence for or against racial/ethnic disparities in profound autism. Notably, the recent CDC prevalence estimates of profound autism found higher proportions of children of color met criteria for profound autism than white children (26). The underlying prevalence rate of autism spectrum disorder in the population at large is not thought to vary by race or ethnicity—the same is thought to be true for profound autism (56, 61). Prior work suggests people of color are less likely to receive timely autism diagnoses than their white peers, which may translate to increased difficulty accessing diagnostic and treatment services (36). There is also evidence to suggest that children of color are more likely to receive a diagnosis of intellectual disability (ID) in lieu of (62) or in addition to (63) a diagnosis of autism compared to their white peers. Clearly, more work examining prevalence rates of profound autism in diverse racial and ethnic groups is needed.

Caregiver interviews highlighted experiences of stigmatization both from society at large, and from medical professionals and other service providers. Regarding the latter, caregivers frequently expressed frustration about the lack of adequate services for their adult children with profound autism. Families expressed frustration finding and maintaining support staff for adults living at home. Difficulties finding respite or other support staff were often reported in tandem with challenging behaviors such as aggression, which corroborates existing qualitative reports on the impact of aggression on experiences of isolation [e.g., (64)]. Families were frustrated by their interactions with medical professionals whom they described as unprepared or unwilling to accommodate the needs of their children. In other words, parents felt that stigmatization towards their adult children with profound autism results in poorer healthcare experiences for their autistic loved ones, as well as limited access to healthcare, residential, and other important services. Similarly in other qualitative reports, caregivers have advocated for all healthcare professionals to receive more autism-specific training, as well as for the use of more person-centered approaches in healthcare, with a particular emphasis on accommodations during visits (65).

Commonly, caregivers reported experiencing stigma in their communities in response to the behaviors of their children. Of particular concern, some families reported feeling they had limited access to their communities and peers for their adult children. Family isolation increases the risks for caregiver burnout and health complications associated with extended caregiving (18, 22). Within the current study, caregivers also frequently reflected on the benefits for their adult children being involved in their communities and with peers. Some caregivers even noted that consistent interaction with community groups, such as church parishes, was integral to reducing their and their children’s stigma experiences over time. Developing opportunities for adults and their family members to increase community and peer engagement remains a critical goal for this population.

Perhaps one of the most frustrating and isolating experiences faced by the parents of profoundly autistic children is the stigmatization they experience within the autism community itself. The heterogeneity of the autism spectrum includes both married college graduates and severely cognitively impaired individuals who will require round the clock supervision for their entire lives. Given these disparate characteristics and the lack of effective labels to parse autism heterogeneity, it is perhaps little wonder that the opinions of families of profoundly autistic individuals and those of some autistic neurodiversity advocates who are capable of leading independent lives frequently differ.

Autistic self-advocates are important stakeholders in debates over policies that affect the autism community—as are the parents and caregivers of profoundly autistic individuals. Importantly, the increasing influence of the former should not come at the expense of the latter. Many individuals with profound autism, by the nature of their intellectual disability and/or limited language capabilities, cannot advocate for themselves. It is vital to acknowledge the invaluable role and enormous efforts of caregivers of children and adults with profound autism as their children’s greatest advocates. Bioethics offers a robust literature on surrogate decision making, and the overwhelming consensus is that family members are the best representatives for incapacitated loved ones, both because they have the deepest understanding of their needs and preferences, and because they care most about their quality of life (66). Yet many neurodiversity proponents have advocated for changes–such as the elimination of words like “treatment,” “severe,” or “challenging behavior” from autism research and clinical practice (67–69)–without meaningful engagement with caregivers nor careful consideration of their articulated concerns for individuals with profound autism. Determining best practices for the most disabled segment of the autism spectrum will require extensive input from the families of profoundly autistic individuals. The consulting of neurodiversity advocates alone is not sufficient. Arguably, the more a particular policy affects profoundly autistic individuals, the more weight should be given to feedback from profoundly autistic individuals’ parents, siblings, and other family members and caregivers.

### Limitations

Our ability to calculate prevalence estimates of profound autism in the present study is inherently limited by the kinds of samples employed in these analyses. The current samples were drawn exclusively from High Income Countries (HICs) and most participants in all six samples were white. The relative lack of racial and ethnic diversity present in the samples described here, as well as discrepancies across samples in how participant diversity was described, limits our ability to adequately examine prevalence rates of profound autism by race and ethnicity. There is also considerable variability in the time periods during which these samples were collected, so the prevalence estimates reported here, particularly for the samples initially collected in the 1990s (AAA and EDX) are likely influenced by cohort effects. Importantly, only two of the six samples (MoBa and SNAP) were population-based, so it is impossible to draw conclusions from this work about the “true” prevalence of profound autism. There were also variations in the measures used across samples to characterize participant IQ and verbal abilities. These measurement differences may have contributed to the different prevalence estimates seen across the six samples.

The caregivers who participated in interviews for the current study are unique in many ways and not representative of the total population of caregivers of adults with profound autism. Although geographically diverse within the United States, most families were white, lived with their adult child, and had adult children diagnosed in early childhood. Further, it is not clear how much the experiences and priorities of families of adult individuals with profound autism overlap with, or are distinct from, the experiences and priorities of families of children with profound autism. Understanding the perspectives of caregivers should be seen as an ongoing effort, and further qualitative exploration into the needs of families of profoundly autistic individuals of all ages, particularly racially diverse and lower SES families, is a priority.

### Future directions

Future work in more racially and ethnic diverse samples is needed to better understand potential disparities that may uniquely impact profoundly autistic individuals of diverse backgrounds and their families. More efforts to establish prevalence estimates of profound autism in LMICs may be especially critical. In LMICs, the relative percentage of autistic individuals who met criteria for profound autism may be higher, given the lack of available assessment and treatment services compared to HICs. In other words, individuals with autism and average or above average IQ and language abilities in LMICs may be less likely to receive an ASD diagnosis and associated services than individuals of similar characteristics in HICs. Accurate and reliable studies on the prevalence of ASD in LMICs are necessary so that health professionals and policy makers can develop strategic plans to meet the needs of autistic individuals (70). A recent review (71) found prevalence studies of autism have only been conducted in 34 countries. Most of the studies included in the review examined the prevalence of autism in HICs, which on average, report higher prevalence estimates of autism than LMICs (72). Access to ASD diagnosis, intervention, and support services is limited in LMICs, many of which do not have sufficient trained healthcare professionals who are familiar with autism to adequately meet service needs (73). Further, in some LMICs, autism and similar developmental conditions are perceived as evidence of demonic possession, curses, or other deeply stigmatizing religious or cultural omens (74, 75). This misinformation regarding the etiology of autism and subsequent stigmatization of people with autism and their families can be a substantial barrier to seeking diagnostic and treatment services in some LMICs.

In short, future work examining the prevalence of ASD, and the prevalence of profound autism specifically, in LMICs should be a priority. Accurate prevalence data in LMICs would underscore the need for policies and funding to improve access to diagnosis and intervention services for autistic individuals and their families. Such research could be also used to improve public awareness of the causes and characteristics of autism, which could in turn mitigate the stigmatization of autistic people and their families. Knowing the prevalence of profound autism specifically would allow policymakers in LMICs to estimate the percentage of the autism population that may need lifelong substantial support—imperative information to prepare public health and service delivery systems to provide adequate care to autistic individuals with the most intensive needs and their families.

Efforts to meaningfully divide up the autism spectrum have persisted for decades and will undoubtedly continue to persist well into the future. As outlined in *The Lancet* Commission, given the huge range seen in the needs and abilities of autistic people, the term “profound autism” was intended to efficiently identify autistic individuals with extensive and often lifelong daily care needs. Future research should also examine the potential utility of describing other subgroups within the autism spectrum. For example, despite not meeting profound autism criteria, some autistic individuals who have fluent language skills and mild or moderate intellectual disability still require substantial daily supports. Still other autistic people may require support in employment, education, and/or other areas of daily life, but are capable of substantial independence when appropriate supports are in place. Careful study is needed to examine and define additional subgroups of the autism spectrum. Ultimately, the goal of any such subgroupings should be to ensure that all individuals with autism and their families receive appropriate services and supports, given their specific abilities and needs.

More work is also needed to understand how stigma impacts access to appropriate diagnostic and treatment services for profoundly autistic individuals. Most individuals with profound ASD will need substantial daily support for much of their lives and will be unable to attain many normative outcomes parents hold for their young children, such as living independently, establishing careers, and having families of their own. Clear and accurate information about the prognosis of profound autism, though that information may be upsetting for parents to hear, is essential for clinicians to communicate to families so that they can prepare financially, mentally, and emotionally for the often-lifelong caregiving responsibilities required for profoundly autistic individuals. But when can clinicians feel confident that an individual meets profound autism criteria, and subsequently share this information with parents and caregivers?

*The Lancet* commission specified that the term profound autism should only be applied to individuals aged eight or older. The rationale for this stipulation was that language fluency and cognitive ability can develop rapidly in early childhood. An autistic child who has very limited speech at age three is unusual, but still may develop many language abilities by age four or five. In contrast, by mid-childhood both language fluency and cognitive ability are relatively stable, and substantial changes are much less likely to occur (44, 76, 77). Nevertheless, Hughes et al. (26) used data on language fluency from as young as age four in their profound autism prevalence estimates and found less than a percentage point difference in their prevalence estimates with language fluency data from age five. More work is needed to establish an empirical basis for profound autism age criteria. Ideally, a data-driven balance should be struck between allowing sufficient time for an individual’s language and cognitive abilities to develop and granting families as much time as possible to prepare for the extensive caregiving responsibilities profoundly autistic individuals require across the life course.

In the United States, all autistic children are entitled by law to appropriate educational support. However, that entitlement ends when autistic individuals age into the world of adult services. The adult services system is plagued by long waiting lists, staffing shortages and frequent staff burnout and turnover [American Network of Community Options and Resources (78)], as well as a fundamental lack of research about the value of different support models (16). Are the dispersed, community-based supports favored by many neurodiversity advocates appropriate for those with profound autism? Might some profoundly autistic adults achieve better outcomes in larger, more structured settings? How do we even define “better outcomes” for those who cannot necessarily articulate their needs and preferences (79, 80)? Additional research is needed to answer these and other pressing questions on how to minimize stigmatization, improve services access, quality of life, and community engagement for profoundly autistic individuals and their families.

## Conclusion

As individuals with autism reach adulthood and avenues for services and community engagement decrease, stigmatization of individuals with profound autism and, notably, their caregivers, may only increase. By calculating the prevalence of profound autism and characterizing experiences of stigmatization and research priorities amongst caregivers of adults with profound autism, the current study enhances our understanding of this vulnerable subgroup of individuals with ASD. Future research should continue to examine the unique needs and stigmatization experiences of this group across the life course.

## Data availability statement

The raw data supporting the conclusions of this article will be made available by the authors, without undue reservation.

## Ethics statement

The studies involving humans were approved by University of California, Los Angeles Office of the Human Research Protection Program, IRB#19–000079. The studies were conducted in accordance with the local legislation and institutional requirements. Written informed consent for participation in this study was provided by the participants’ legal guardians/next of kin.

## Author contributions

EC: Conceptualization, Writing – original draft, Writing – review & editing. JM: Conceptualization, Writing – original draft, Writing – review & editing. AL: Writing – original draft, Writing – review & editing. MG: Writing – original draft, Writing – review & editing. SS: Conceptualization, Data curation, Writing – review & editing. CL: Conceptualization, Data curation, Funding acquisition, Supervision, Writing – review & editing.
