# Corrigendum: Understanding profound autism: implications for stigma and supports

**DOI:** 10.3389/fpsyt.2024.1396556

**Published:** 2024-06-13

**Authors:** Elaine B. Clarke, James B. McCauley, Amy Lutz, Marina Gotelli, Stephen J. Sheinkopf, Catherine Lord

**Affiliations:** ^1^ Department of Psychiatry and Biobehavioral Sciences, University of California, Los Angeles, Los Angeles, CA, United States; ^2^ Department of Psychology, St. Mary’s College of California, Moraga, CA, United States; ^3^ Department of History and Sociology of Science, University of Pennsylvania, Philadelphia, PA, United States; ^4^ Fundación Brincar por un Autismo Feliz, Buenos Aires, Argentina; ^5^ Consejo Nacional de Investigaciones Científicas Y Técnicas, Buenos Aires, Argentina; ^6^ Thompson Center for Autism and Neurodevelopment, University of Missouri, Columbia, MO, United States

**Keywords:** autism spectrum disorder, profound autism, stigma and awareness, prevalence, mixed method, qualitative interview analysis, autistic adults

In the published article, there was an error in **Table 2**, *Profound Autism Prevalence Estimates by Sample, Gender, and Race*, as published. We have identified several typographical errors in the original Lancet Commission, from which the statistics in **Table 2** for the EDX sample were derived. The corrected **Table 2**, *Profound Autism Prevalence Estimates by Sample, Gender, and Race* and its caption, appear below.

**Table 2:** Profound autism prevalence estimates by sample, gender, and race.

Sample		Profound Autism Prevalence						
		Overall	Gender		Race			
			Male	Female	White	People of Color*		
**Adolescents and Adults with Autism (AAA)**		57% (49 – 64%)	54% (45 –62%)	70% (51 – 84%)	52% (42 – 61%)	69% (55 – 81%)		
**Early Diagnosis Cohort (EDX)**		48% (37 – 58%)	4% (0 – 11%)	23% (10 – 36%)	34% (27 – 42%)	70% (55 – 81%)		
**Special Needs and Autism Project (SNAP)**	**Unweighted**	23% (16 – 30%)	22% (16 – 30%)	25% (7 – 52%)	22% (16 – 30%)	20% (10 – 37%)		
	**Weighted**	20% (10 – 36%)	21% (10 – 39%)	15% (3 – 50%)	25% (3 – 65%)	11% (1 – 55%)		
**QUEST**	**Unweighted**	31% (21 – 43%)	26% (14 – 41%)	14% (7 – 26%)	**White**	**Black**	**Multi**	**Other**
					29% (16 – 30%)	45% (24 – 68%)	10% (0 – 45%)	33% (7 – 70%)
	**Weighted**	18% (11 – 28%)	38% (22 – 56%)	38% (23 – 56%)	15% (7 – 29%)	30% (14 – 55%)	6% (0 – 44%)	23% (4 – 69%)
**Rhode Island Consortium of Autism Research and Treatment (RI-CART)**		11% (8 – 15%)	14% (10 – 19%)	9% (4 – 17%)	**White**	People of Color*		
					13% (9 – 18%)	16% (11 – 22%)		
**Norwegian Mother, Father, and Child Cohort (MoBa)**		18% (12 – 24%)	17% (12 –24%)	45% (28 – 63%)	**Caregiver Native Language^†^**			
					**Native Norwegian Speaker**	**Non-Native Norwegian Speaker**		
					23% (17 – 30%)	22% (11 – 39%)		

^*^Due to limited numbers of racially and ethnically diverse participants (AAA, SNAP) or the majority of racially and ethnically diverse participants belonging to a single racial/ethnic group (Black, EDX), racial and ethnic prevalence estimates for these samples were collapsed into binary categories. ^†^For the MoBA sample only, caregiver native language was used as a proxy for measuring racial and ethnic diversity.

In the published article, there was an error the **Results**, *United States Samples Prevalence Estimates*, Paragraph 1. In the original Lancet paper, from which **Table 2** and some of the **Results** section were derived, the proportions of individuals with profound autism in the EDX sample were incorrect. This sentence previously stated:

“The proportion of individuals meeting one or both criteria for profound autism criteria was 48% (95% CI 37–58%) in the EDX sample. A higher proportion of females in EDX met profound autism criteria than males, although confidence ranges overlapped (23% vs. 4%, see **Table 2** for confidence intervals). Moreover, a higher proportion of participants of color met criteria for profound autism in the EDX sample compared to white participants (70% vs. 34%).”

The corrected sentence appears below:

“The proportion of individuals meeting one or both criteria for profound autism criteria was 57% (95% CI 49–64%) in the EDX sample. A higher proportion of females in EDX met profound autism criteria than males, although confidence ranges overlapped (70% vs. 54%, see **Table 2** for confidence intervals). Moreover, a higher proportion of participants of color met criteria for profound autism in the EDX sample compared to white participants (69% vs. 52%).”

The authors apologize for these errors and state that this does not change the scientific conclusions of the article in any way. The original article has been updated.

